# Enhanced progression of human prostate cancer PC3 cells induced by the microenvironment of the seminal vesicle

**DOI:** 10.1038/sj.bjc.6604169

**Published:** 2008-01-08

**Authors:** M Kumano, H Miyake, T Kurahashi, K Yamanaka, M Fujisawa

**Affiliations:** 1 Department of Surgery, Division of Urology, Kobe University Graduate School of Medicine, 7-5-1 Kusunoki-cho, Chuo-ku, Kobe 650-0017, Japan

**Keywords:** prostate cancer, invasion, seminal vesicle, transforming growth factor-*β*_1_, urokinase-type plasminogen activator

## Abstract

The objective of this study was to characterise the mechanism mediating the prostate cancer progression induced by the microenvironment of seminal vesicle (SV). The invasive potential of PC3 cells significantly increased after treatment with extract from SV of NOD/SCID mouse. Among several growth factors and cytokines that were present in the SV extract, transforming growth factor-*β*_1_ (TGF-*β*_1_) significantly enhanced the invasive potential of PC3 cells; however, the additional treatment with neutralising antibody against TGF-*β*_1_ suppressed the enhanced invasive potential induced by the SV extract. Changes in the invasive potential in PC3 cells after treatment with the SV extract and/or TGF-*β*_1_ were in proportion to those in the production of urokinase-type plasminogen activator (uPA) by PC3 cells. Tumour growth as well as the incidence of lymph node metastasis in NOD/SCID mice after the injection of PC3 cells into the SV were significantly greater than those after the injection into the prostate. These findings suggest that the microenvironment of SV enhances the progression of prostate cancer through a stimulated invasive potential, and that enhanced uPA production in prostate cancer cells induced by TGF-*β*_1_ could therefore be one of the most important mechanisms involved in the progression of prostate cancer after SV invasion.

Invasion of prostate cancer cells into the seminal vesicle (SV) is an adverse prognostic factor in patients undergoing radical prostatectomy. Contemporary series analysing outcomes of radical prostatectomy reported that biochemical recurrence occurred in more than 50% of patients with SV invasion (Sofer *et al*, 2003; Bloom *et al*, 2004). However, SV invasion has been shown to lack a systematic relationship with other potential pathological factors indicating a poor prognosis, and there has not been any independent prognostic predictor in patients with SV invasion (Sofer *et al*, 2003; Masterson *et al*, 2005). These findings suggest that adverse features of prostate cancer with SV invasion may be due to an acquired aggressive phenotype rather than ‘volume effect’ as a result of disease progression.

The outcome of cancer progression depends on multiple interactions of cancer cells with various host factors; that is, the interaction of tumour cells with the organ environment modulates the tumorigenic properties by regulating their phenotypes, such as cell proliferation, motility and invasion (Fidler, 1990). A number of studies have demonstrated significant effects of organ microenvironment on the malignant potentials in several types of cancer cells, including prostate cancer, using *in vivo* experimental models (Gohji *et al*, 1997; Sato *et al*, 1997; Alencar *et al*, 2005). For example, Gohji *et al* reported that human renal cancer cells implanted in the subcutis of nude mice produced local nonmetastatic tumours, whereas the same cells orthotopically implanted in the kidney resulted in the formation of local tumours and metastases to the lungs. Furthermore, they clarified the important role of proteolytic enzymes whose production is influenced by the organ microenvironment, in the progression of implanted renal cell carcinoma cells (Gohji *et al*, 1997).

To date, the molecular mechanism mediating disease progression following SV invasion has remained largely unknown, and there is no study analysing whether the malignant phenotype of prostate cancer cells is affected by the microenvironment of SV. We, therefore, developed an implantation model of human prostate cancer cells to the SV of immunodeficient mice, resulting in systemic disease progression *in vivo*, and investigated the mechanism underlying the modulation of malignant potential of human prostate cancer cells induced by the microenvironment of SV.

## MATERIALS AND METHODS

### Reagents

Recombinant human transforming growth factor-*β*_1_ (TGF-*β*_1_), basic fibroblast growth factor, hepatocyte growth factor, platelet-derived growth factor, granulocyte colony-stimulating factor, anti-human TGF-*β*_1_ antibody, and anti-human epidermal growth factor (EGF) antibody were from R&D Systems (Minneapolis, MN, USA). Recombinant human EGF, granulocyte monocyte colony-stimulating factor, tumour necrosis factor-*α*, interkeukin-1*β*, interleukin-6, fibronectin, and anti-rat *β*-tubulin antibody were from Chemicon International (Temecula, CA, USA). Anti-human urokinase-type plasminogen activator (uPA) antibody and quantitative sandwich enzyme immunoassay kit for human uPA were from American Diagnostica (Greenwich, CT, USA). Horseradish peroxidase-conjugated anti-mouse IgG antibody was from Amersham Life Science (Arlington Heights, IL, USA). Biotinylated goat anti-mouse IgG was from Vector Laboratories (Burlingame, CA, USA).

### Tumour cell line

PC3, derived from human prostate cancer, was purchased from the American Type Culture Collection (Rockville, MD, USA). Cells were maintained in DMEM (Life Technologies Inc., Gaithersburg, MD, USA) supplemented with 5% heat-inactivated fetal calf serum.

### Preparation of extracts from the SV and prostate

After 12-week-old nonobese diabetic/severe combined immunodeficient (NOD/SCID) mice (CLEA Japan Inc., Tokyo, Japan) were killed, SV and prostate were harvested, washed with PBS, and disrupted using a sonicator (Ultrasonic Systems Inc., Haverhill, MA, USA). Following centrifugation of the respective extracts of SV and prostate, each supernatant was stored at −80°C until used.

### *In vitro* cell growth assay

The effects of extract from the SV or prostate on the *in vitro* growth of PC3 cells were assessed using MTT (Sigma Chemical Co., St Louis, MO, USA) as described previously (Yamanaka *et al*, 2005). Briefly, 1 × 10^4^ cells were seeded in each well of 96-well microtitre plates and allowed to attach overnight. Cells were then treated with various concentrations of either SV or prostate extract diluted with serum-free DMEM/F12. After 48 h of incubation, 20 *μ*l of 5 mg ml^−1^ MTT in PBS was added to each well, followed by incubation for 4 h at 37°C. The formazan crystals were dissolved in DMSO. The optical density was determined with a microculture plate reader (Becton Dickinson Labware, Lincoln Park, NJ, USA) at 540 nm. Absorbance values were normalised to the values obtained for vehicle-treated cells. Each assay was performed in triplicate.

### *In vitro* tumour cell invasion assay

Tumour cell invasion was measured using cell invasion assay kit (Chemicon) as described previously (Miyake *et al*, 1999a). Briefly, we used polycarbonate filters with a pore size of 8 *μ*m coated with basement membrane Matrigel. The coated filters were placed in Boyden chambers, in the upper compartment of which 1 × 10^5^ cells were suspended in serum-free DMEM/F-12 followed by treatment with extract from the SV or prostate and/or growth factor or cytokine and in the lower compartment of which 25 *μ*g ml^−1^ fibronectin diluted with serum-free DMEM/F-12 were added as a chemoattractant. After 48 h incubation at 37°C, cells on the top side of the filter were removed, and cells that had migrated and invaded the Matrigel through the filter and attached to the bottom of the membrane were stained with crystal violet stain solution. The crystal violet stain solution was eluted with 10% acetic acid extraction buffer and transferred to wells of a 96-well microtitre plate, and the absorbance was read with a microculture plate reader (Becton Dickinson Labware) at 540 nm. Absorbance values were normalised by the values obtained for the vehicle-treated cells. Similarly, cell motility was also assessed using the Boyden chambers without matrigel. Each assay was performed in triplicate.

### Measurement of uPA levels in conditioned media

The concentrations of uPA in conditioned media were determined using a quantitative sandwich enzyme immunoassay kit for human uPA as described previously (Miyake *et al*, 1999b). Briefly, PC3 cells were seeded in each well of 96-well microtitre plates and allowed to attach overnight. Cells were then treated with extract from the SV, TGF-*β*_1_, and/or anti-TGF-*β*_1_ antibody diluted with serum-free DMEM/F-12. After 48 h of incubation, serum-free DMEM/F-12 was collected. For each analysis, 100 *μ*l of conditioned media were added to microtitre plates coated with a purified polyclonal antibody against human uPA. Bound uPA was detected by an additional biotinylated anti-uPA antibody. After the addition of streptavidin-conjugated horseradish peroxidase, peroxidase-mediated conversion of 3,3′,5,5′-tetramethylbenzene was measured with a microculture plate reader (Becton Dickinson Labware) at 450 nm. Each assay was performed in triplicate.

### Western blot analysis

Western blot analysis was used to evaluate the expression level of uPA protein in PC3 cells after treatment with extract from the SV and/or anti-TGF-*β*_1_ antibody as described previously (Miyake *et al*, 1999a). Briefly, samples containing equal amounts of protein (15 *μ*g) from lysates of the cultured PC3 cells were electrophoresed on an SDS-polyacrylamide gel and transferred to a nitrocellulose filter. The filters were blocked in PBS containing 5% nonfat milk powder at 4°C overnight and then incubated for 1 h with a 1 : 400-diluted anti-human uPA mouse antibody or 1 : 10 000-diluted anti-rat *β*-tubulin mouse antibody. The filters were then incubated for 30 min with horseradish peroxidase-conjugated anti-mouse IgG antibody, and specific proteins were detected using an enhanced chemiluminescence western blotting analysis system (Amersham Life Science).

### Assessment of *in vivo* tumour growth

Male NOD/SCID mice, 10- to 12-week-old, (CLEA Japan Inc.) were housed in a controlled environment at 22°C on a 12-h light and 12-h dark cycle. Animals were maintained in accordance with the National Institutes of Health Guide for the Care and Use of Laboratory Animals. Each experimental group consisted of 10 mice. PC3 cells were trypsinised, washed twice with PBS, and 5 × 10^5^ cells suspended in 20 *μ*l of PBS were directly injected into the SV or the dorsal love of the prostate under the prostatic capsule. Eight weeks after the injection of tumour cells, the mice were killed and the presence of metastasis was macroscopically examined in all abdominal organs, and the weight of each tumour formed in the SV or prostate was measured.

### Statistical analysis

Differences between the two groups were compared using the *χ*^2^-test, unpaired *t*-test or Mann–Whitney *U*-test. All statistical calculations were performed using Statview 5.0 software (Abacus Concepts Inc., Berkley, CA, USA), and *P*-values <0.05 were considered significant.

## RESULTS

### Changes in the malignant phenotype of PC3 cells induced by extract from the SV or prostate

**Figure 1:**
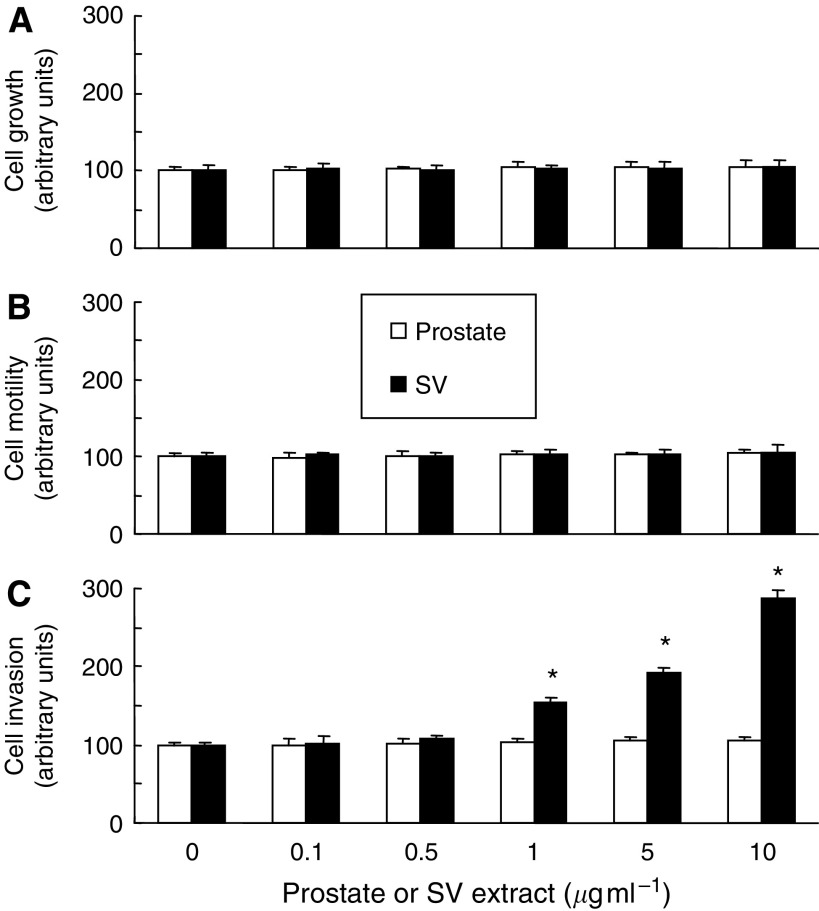
Effects of treatment with extract of either prostate or seminal vesicle (SV) on malignant phenotypes, including cell growth, motility and invasion, in human prostate cancer PC3 cells. (**A**) PC3 cells were treated with various concentrations of the prostate or SV extract diluted with serum-free DMEM/F12. After 48 h of incubation, the number of viable cells was determined by the MTT assay. Columns, mean of three independent experiments; bars, s.d. (**B**) PC3 cells seeded at 1 × 10^5^ per well in Boyden chambers were treated with various concentrations of the prostate or SV extract diluted with serum-free DMEM/F12. Chambers were incubated for 48 h in serum-free DMEM/F12, and then cells that had migrated to the lower surface of filters were stained with crystal violet stain solution. After the elution of crystal violet, the absorbance value in each well was measured with a microculture plate reader. Columns, mean of three independent experiments; bars, s.d. (**C**) PC3 cells seeded at 1 × 10^5^ per well in Boyden chambers were treated with various concentrations of the prostate or SV extract diluted with serum-free DMEM/F12. Chambers were incubated for 48 h, and then cells that had migrated to the lower surface of filters through reconstituted basement membrane Matrigel were stained with crystal violet stain solution. After the elution of crystal violet, the absorbance value in each well was measured with a microculture plate reader. Columns, mean of three independent experiments; bars, s.d. ^*^, differs from control (*P*<0.01).

We initially evaluated the effects of SV or prostate extract on the malignant potential of PC3 cells. As shown in Figure 1, neither the SV or prostate extract had any impact on cell growth or motility of PC3 cells. However, despite the lack of significant effect of prostate extract on the invasive potential of PC3 cells, treatment of PC3 cells with SV extract increased the invasive potential in a dose-dependent manner.

### Influence of growth factors and cytokines on the invasive potential of PC3 cells

**Figure 2:**
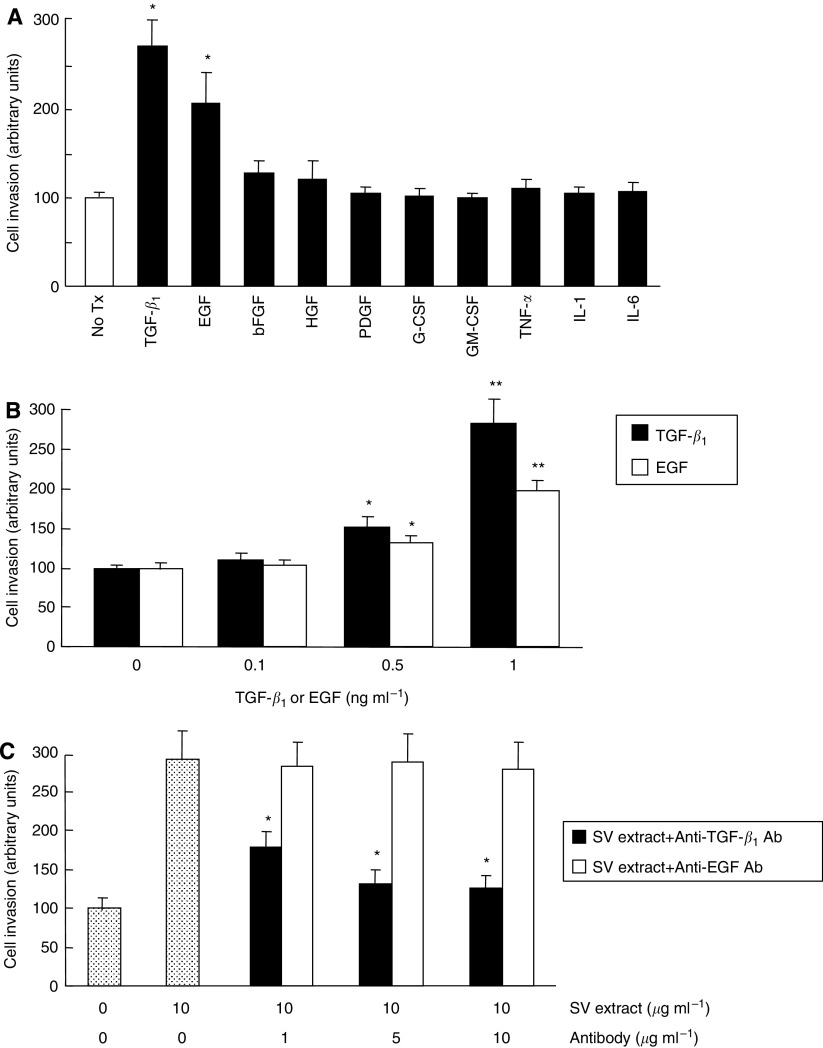
Effects of treatment with growth factors and cytokines on invasive potential in human prostate cancer PC3 cells. (**A**) PC3 cells seeded at 1 × 10^5^ per well in Boyden chambers were treated with 1 ng ml^−1^ transforming growth factor-*β*_1_ (TGF-*β*_1_), 1 ng ml^−1^ epidermal growth factor (EGF), 10 ng ml^−1^ basic fibroblast growth factor (bFGF), 10 ng ml^−1^ hepatocyte growth factor (HGF), platelet-derived growth factor (PDGF), 10 ng ml^−1^ granulocyte colony-stimulating factor (G-CSF), 10 ng ml^−1^ granulocyte monocyte colony-stimulating factor (GM-CSF), 100 U ml^−1^ tumour necrosis factor-*α* (TNF-*α*), 100 U ml^−1^ interkeukin-1*β* (IL-1*β*) or 10 ng ml^−1^ interleukin-6 (IL-6) diluted with serum-free DMEM/F12. Chambers were incubated for 48 h, and then cells that had migrated to the lower surface of filters through reconstituted basement membrane Matrigel were stained with crystal violet stain solution. After the elution of crystal violet, the absorbance value in each well was measured with a microculture plate reader. No Tx, untreated cells. Columns, mean of three independent experiments; bars, s.d. ^*^, differs from control (*P*<0.01). (**B**) PC3 cells seeded at 1 × 10^5^ per well in Boyden chambers were treated with various doses of TGF-*β*_1_ or EGF diluted with serum-free DMEM/F12. Chambers were incubated for 48 h, and then cells that had migrated to the lower surface of filters through reconstituted basement membrane Matrigel were stained with crystal violet stain solution. After the elution of crystal violet, the absorbance value in each well was measured with a microculture plate reader. Columns, mean of three independent experiments; bars, s.d. ^**^ and ^*^, differs from control (*P*<0.01 and *P*<0.05, respectively). (**C**) PC3 cells seeded at 1 × 10^5^ per well in Boyden chambers were treated with 10 *μ*g ml^−1^ seminal vesicle extract and various doses of anti-TGF-*β*_1_ or anti-EGF antibody diluted with serum-free DMEM/F12. Chambers were incubated for 48 h, and then cells that had migrated to the lower surface of filters through reconstituted basement membrane Matrigel were stained with crystal violet stain solution. After the elution of crystal violet, the absorbance value in each well was measured with a microculture plate reader. Columns, mean of three independent experiments; bars, s.d. ^*^, differs from control (*P*<0.01).

To identify candidate factor responsible for the enhanced invasive potential of PC3 cells induced by SV extract, the abilities of 10 kinds of growth factors or cytokines to increase the invasive potential of PC3 cells were evaluated. Of the 10 factors tested, TGF-*β*_1_ and EGF significantly stimulated the invasive potential of PC3 cells (Figure 2A). Furthermore, treatment with both TGF-*β*_1_ and EGF increased in the invasive potential of PC3 cells in dose-dependent manners (Figure 2B). We then examined whether antibody against TGF-*β*_1_ or EGF suppresses the enhanced invasive potential of PC3 cells induced by the SV extract. As shown in Figure 2C, anti-TGF-*β*_1_ antibody inhibited the invasive potential of PC3 cells in a dose-dependent manner, while the invasive ability of PC3 cells stimulated by SV extract was not affected by anti-EGF antibody. These results suggest that TGF-*β*_1_ in seminal plasma may be involved in the enhanced invasive potential of PC3 cells induced by the SV extract.

### Regulation of uPA production in PC3 cells by TGF-*β*_1_

**Figure 3:**
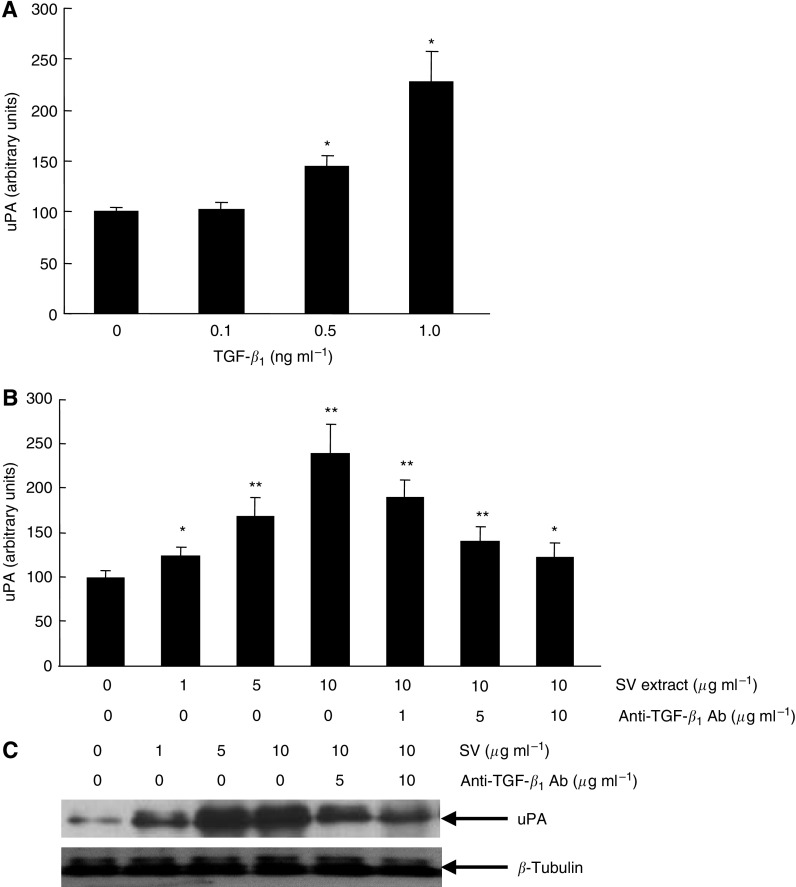
Regulation of urokinase-type plasminogen activator production in human prostate cancer PC3 cells by transforming growth factor-*β*_1_ (TGF-*β*_1_). (**A**) PC3 cells were treated with various concentrations of TGF-*β*_1_ diluted with serum-free DMEM/F12. After 48 h of incubation, serum-free DMEM/F12 was collected, and the concentration of uPA in each sample was determined with a quantitative sandwich enzyme immunoassay kit for human uPA. Columns, mean of three independent experiments; bars, s.d. ^*^, differs from control (*P*<0.01). (**B**) PC3 cells were treated with various concentrations of seminal vesicle (SV) extract and anti-TGF-*β*_1_ antibody diluted with serum-free DMEM/F12. After 48 h of incubation, serum-free DMEM/F12 was collected, and the concentration of uPA in each sample was determined with a quantitative sandwich enzyme immunoassay kit for human uPA. Columns, mean of three independent experiments; bars, s.d. ^**^ and ^*^, differs from control (*P*<0.01 and *P*<0.05, respectively). (**C**) PC-3 cells were treated with various concentrations of SV extract and/or anti-TGF-*β*_1_ antibody diluted with serum-free DMEM/F12. After 48 h of incubation, protein was extracted from culture cells, and uPA and *β*-tubulin protein levels were analysed by western blotting.

To investigate the mechanism mediating the stimulation of invasive potential of PC3 cells by treatment with TGF-*β*_1_, we analysed the role of uPA, one of the most important proteolytic enzymes involved in tumour cell invasion (Festuccia *et al*, 1998), in this process. Treatment of PC3 cells by TGF-*β*_1_ resulted in a dose-dependent increase in uPA production released in the culture medium (Figure 3A). Furthermore, the SV extract also induced increased uPA production by PC3 cells in a dose-dependent manner; however, this stimulated production of uPA by treatment with the SV extract was significantly inhibited by additional treatment with anti-TGF-*β*_1_ antibody (Figure 3B). Western blot analysis was used to measure changes in the expression levels of uPA protein in PC3 cells following treatment with SV extract and/or anti-TGF-*β*_1_ antibody. As shown in Figure 3C, uPA protein expression in PC3 cells was enhanced by treatment with SV extract in a dose-dependent manner, whereas treatment with anti-TGF-*β*_1_ antibody resulted in the suppression of enhanced uPA protein expression by the SV extract in PC3 cells.

### Disease progression following the injection of PC3 cells into the SV in NOD/SCID mice

To compare the effects of organ microenvironment between SV and prostate on the disease progression of PC3 tumours *in vivo*, we injected PC3 cells into either the SV or prostate in NOD/SCID mice. The mice were killed 8 weeks after the tumour cell injection, during which we found that the weight of tumours in mice receiving SV injection was significantly greater than that in mice receiving prostate injection. Furthermore, the incidence of retroperitoneal lymph node metastases in mice receiving SV injection was significantly higher than that in mice receiving prostate injection (Table 1). In addition, haemorrhagic ascites was observed only in the mice following SV injection.

## DISCUSSION

Although SV invasion has been regarded as one of the most potent factors related to an adverse prognosis in patients undergoing radical prostatectomy (Sofer *et al*, 2003; Bloom *et al*, 2004), the molecular mechanism mediating the progression of prostate cancer following the invasion of cancer cells into the SV remains largely unknown. To date, a number of studies have demonstrated a significant impact of organ microenvironment on disease progression of various types of human malignant tumours (Gohji *et al*, 1997; Sato *et al*, 1997; Alencar *et al*, 2005); however, there have not been any studies investigating the significance of the SV microenvironment as a factor influencing the progression of prostate cancer. In this study, therefore, we focused on the role of microenvironment of the SV, and evaluated its effects on changes in malignant phenotypes of human prostate cancer PC3 cells both *in vitro* and *in vivo*.

It was initially examined whether the SV or prostate extract influences the malignant potential of PC3 cells, and demonstrated that despite the lack of a significant effect of prostate extract, the invasive potential of PC3 cells was markedly enhanced by SV extract. To identify the factors in SV extract mediating the stimulation of invasive potential, we examined the effects of 10 different growth factors or cytokines that have been shown to be abundantly present in seminal plasma (Matalliotakis *et al*, 1998; Robertson, 2005) and/or associated with the invasion of prostate cancer cells (Barton *et al*, 2001; Bindukumar *et al*, 2005). Of the 10 factors examined in this study, TGF-*β*_1_ and EGF were found to significantly stimulate the invasive potential of PC3 cells; however, anti-TGF-*β*_1_ antibody, but not anti-EGF antibody, suppressed the enhanced invasive potential of PC3 cells induced by SV extract. These findings suggest that TGF-*β*_1_ may, at least in part, be involved in the increased invasive potential of PC3 cells induced by SV extract.

It is of interest to clarify the mechanism by which TGF-*β*_1_ induces the increased invasive potential of PC3 cells. Several previous studies have shown that TGF-*β*_1_ enhances the secretion of proteolytic enzymes in prostate cancer cells, which helps degrade the connective tissue extracellular matrix and basement membrane components (Festuccia *et al*, 2000; Unlu and Leake, 2003). Among these enzymes involved in tumour cell invasion, uPA is one of the most predominant factors involved in the disease progression of malignant tumours (Choong and Nadesapillai, 2003). In prostate cancer as well, accumulating evidence strongly suggests the important role of uPA in the disease progression of prostate cancer (Pulukuri *et al*, 2005; Usher *et al*, 2005; Shariat *et al*, 2007). For example, Pulukuri *et al* (2005) reported that RNA interference-directed knockdown of uPA and its receptor in PC3 cells significantly reduced tumour cell viability and invasion, and ultimately resulted in the induction of apoptotic cell death. Considering these findings, in this study, we analysed the TGF-*β*_1_-induced stimulation of invasive potential in PC3 cells focusing on the role of uPA. Interestingly, treatment of PC3 cell with TGF-*β*_1_ enhanced their secretion of uPA in a dose-dependent manner. In addition, inhibition of TGF-*β*_1_ activity in the SV extract resulted in the suppression of uPA production in PC3 cells, which was proportional to their invasive potential. Collectively, these results indicated the potential role of uPA in TGF-*β*_1_-mediated enhanced invasive potential of PC3 cells.

To compare the different effects of organ microenvironment between the SV and prostate on disease progression *in vivo*, we directly injected PC3 cells into the SV or prostate in NOD/SCID mice. A number of studies have demonstrated that cancer cells, including prostate cancer, can achieve favourable environments for disease progression in anatomically relevant (i.e., orthotopic) organs (Gohji *et al*, 1997; Sato *et al*, 1997; Alencar *et al*, 2005). In this study as well, lymph node metastases was observed in some mice following injection of PC3 cells into the prostate as described previously (Saffran *et al*, 2001); however, disease progression in mice following the SV injection of PC3 cells was more prominent than that in mice following intraprostatic injection. Furthermore, we performed *in vivo* experiments injecting androgen-dependent human prostate cancer LNCaP cells into the SV or the prostate of NOD/SCID mice, and demonstrated that tumour growth as well as the incidence of lymph node metastasis after the injection of LNCaP cells into the SV were significantly greater than those after the injection into the prostate (data not shown). To our knowledge, this is the first study clearly showing that SV rather than the orthotopic organ (i.e., prostate) provides a stimulating environment for the progression of prostate cancer cells.

Here, we would like to emphasise several limitations of this study. First, the phenomenon of uPA induction by TGF-*β*_1_ may not be entirely responsible for the enhanced invasive potential of PC3 cells by treatment with SV extract; that is, other molecules present in the SV may be involved in promoting the invasive potential. In addition, different mechanisms associated with the microenvironment of the SV, such as the regulated production of proteolytic enzymes by organ-specific fibroblasts (Gohji *et al*, 1997), may have a significant impact on the disease progression following the injection of PC3 cells into the SV. In fact, despite the lack of a stimulatory effect of SV extract on the proliferation of PC3 cells, injection of PC3 cells into the SV resulted in the enhanced growth of PC3 tumours, suggesting the involvement of a mechanism other than the SV extract-induced enhanced invasive potential of PC3 cells in the disease progression of PC3 tumours *in vivo*. It is also a limitation that the findings presented in this study may not be uniformly true for a wide variety of cancer cells, considering a divergent function of TGF-*β*_1_ in modulating the malignant phenotype of cancer cells (Desruisseau *et al*, 1996; Tuxhorn *et al*, 2002). Finally, we could not show *in vivo* data supporting the hypothesis of this study; that is, there were no characteristic findings on histological examinations of both tumours developed after the injection of PC3 cells into the SV and the prostate, such as those suggesting different metastatic potentials, and both tumours were shown to exhibit strong uPA expression by immunohistochemical staining (data not shown), which may be due to the originally high expression level of uPA in PC3 cells, resulting in the lack of evident difference in uPA expression between tumours in the SV and the prostate. Accordingly, to enhance the reliability of our findings, the outcomes of this study should be confirmed using different kinds of human prostate cancer model systems.

In conclusion, the findings presented in this study suggest that the microenvironment of SV enhances the progression of prostate cancer through a stimulated invasive potential, and that enhanced uPA production in prostate cancer cells induced by TGF-*β*_1_, which is abundantly present in seminal plasma, could therefore be one of the most important mechanisms involved in the progression of prostate cancer following SV invasion. To address the functional impact of TGF-*β*_1_-induced uPA production on the progression of prostate cancer in the microenvironment of SV, it would be absolutely necessary to perform further experiments characterising changes in malignant phenotype of PC3 cells both *in vitro* and *in vivo* before and after the inactivation of TGF-*β*_1_ and/or uPA.

## Figures and Tables

**Table 1:** Comparison of disease progression *in vivo* following the injection of PC3 cells into the prostate or SV

	**Prostate injection**	**SV injection**	***P*-value**
Incidence of lymph node metastasis (%)^a^	2/10 (20)	7/10 (70)	<0.05
Incidence of haemorrhagic ascites (%)^b^	0/10 (0)	4/10 (40)	<0.05
Weight of the primary tumor (mg)^c^	22.8±8.7	40.7±10.6	<0.01

SV=seminal vesicle.

a No. of mice with lymph node metastases/no. of injected mice.

b No. of mice with haemorrhagic ascites/no. of injected mice.

c Mean±s.d.
